# Phosphorus Alters Starch Morphology and Gene Expression Related to Starch Biosynthesis and Degradation in Wheat Grain

**DOI:** 10.3389/fpls.2017.02252

**Published:** 2018-01-12

**Authors:** Runqi Zhang, Cheng Li, Kaiyong Fu, Chao Li, Chunyan Li

**Affiliations:** Xinjiang Production and Construction Group, The Key Laboratory of Oasis Eco-Agriculture, College of Agriculture, Shihezi University, Shihezi, China

**Keywords:** biosynthesis, degradation, phosphorus, starch, wheat

## Abstract

Phosphorus is an essential plant macronutrient which profoundly affects the yield and quality of wheat starch. In this study, scanning electron microscopy showed that P fertilizer amount (0, 46, and 92 kg P ha^−1^) had no significant effect on the shape of starch granules in wheat (cv. Xindong 20) grain. However, confocal laser scanning microscopy with 3-(4-carboxybenzoyl) quinoline-2-carboxaldehyde and methanolic merbromin stains indicated that P amount influenced the microstructure of the starch granules. Starch granules from the 46 kg P ha^−1^ treatment released significantly more reducing sugars than those from the 0 and 92 kg P ha^−1^ treatments during digestion with alpha-amylase and amyloglucosidase digestion. Phosphorus application (especially the 46 kg P ha^−1^ treatments) significantly increased the relative expression of genes related to starch synthesis (especially during early to mid-grain filling) and starch degradation (especially during mid- and late grain filling). Phosphorus application also increased the transcript abundance of amylase genes at the periphery of the endosperm. We propose that P application, especially the 46 kg P ha^−1^ treatment, enhanced channels in wheat starch granules. These channels facilitated the transport of substances required for starch biosynthesis, thus increasing starch accumulation in wheat endosperm. These results provide insight into the potential mechanisms through which P influences the microstructure and biosynthesis of wheat starch.

## Introduction

Phosphorus (P) is one of the main limiting factors for plant growth in natural ecosystems. The application of P fertilizer is often essential for crop production (Buchanan et al., 2004). Plants generally take up soil P in its inorganic forms. However, 50–80% of the total P in agricultural soils exists as organic phosphate, which is biologically unavailable (Wang et al., 2009). Wheat is grown under P-poor as well as P-rich conditions. In their efforts to maximize field, farmers often over apply P fertilizer. The fertilization rates in some areas are several times greater than the amount required by wheat. This can create serious environmental problems (Tang et al., 2008; Wang et al., 2017). It should also be noted that rock phosphate is a non-renewable resource that is being depleted (Wang et al., 2017). More understanding is needed about how P affects wheat yield and quality.

The main component of wheat endosperm is starch, which accounts for ~70% of grain dry weight. Many enzymes are involved in starch biosynthesis including granule-bound starch synthase (GBSS), adenosine diphosphate glucose pyrophosphorylase (AGPase), starch branching enzyme (SBE), starch debranching enzyme (DBE), and soluble starch synthase (SSS). The AGPase is activated by 3-phosphoglycerate and is inhibited by inorganic P in leaves. The activity of AGPase can be changed by altering the ratio of 3-phosphoglycerate and inorganic P, which in turn regulates starch synthesis (MacDonald and Strobel, 1970).

Starch molecules are deposited as semi-crystalline structures in starch granules (Tang et al., 2006). Wheat starch can be classified as either large A-type granules (>10 μm diam.) or small B-type granules (<10 μm diam.). The two types of granules are differentiated by their morphological and chemical characteristics (Yu et al., 2015).

Pores and radial, tube-like channels have been observed at the surfaces of starch granules in wheat (Kim and Huber, 2008), corn, and sorghum (Huber and Bemiller, 2000). It has been hypothesized that the pores are not just surface features but might be openings to channels that provide access to the interior of starch granules (Huber and Bemiller, 2000). Starch granule architecture suggests that the pores and channels may be loosely assembled zones in “defective” blocklets (Tang et al., 2006). Pores and channels within starch granules have important influence on granule accessibility to reagents. This means that pores and channels can influence the reactivity of starch when it is chemically modified for specific purposes in industry (Huber and BeMiller, 1997; Han et al., 2005). Enzymatic digestion is also greatly influenced by pores and channels within native starch granules. Maize starch, which contains pores and channels, is more susceptible to enzymatic hydrolysis than potato starch (Han et al., 2005), which does not have pores (Fannon et al., 1992a). It is possible that starch granule channels can be manipulated to improve digestibility or alter the chemical characteristics of starch (Han et al., 2005). However, much more information is needed about these channels and their biological origin.

Environmental conditions such as high temperature (Li et al., 2017) and drought stress (Li et al., 2015) can alter the pits and channels in wheat starch granules. Commuri and Jones (1999) postulated that pitting is induced by imbalances in starch hydrolase and synthase, resulting in premature autolysis. Based on the protein constituents of channels in normal maize starch granules, it has been proposed that the channels are remnants of amyloplast microtubules and may facilitate starch polymer and granule biosynthesis (Fannon et al., 2004; Benmoussa et al., 2010).

Previous research in our laboratory indicated that the P fertilizer can also significant affect the characteristics of wheat starch, especially the presence of “pin holes” within starch granules (Li et al., 2013). The objective of this study was to increase understanding about the influence of P fertilizer on starch biosynthesis and granule structure in wheat.

## Materials and methods

### Plant material and cultivation

The study was conducted at the Shihezi University Experimental Farm, Shihezi, China (44°17′ N, 86°03′ E) from October 2014 to June 2015. The soil at the site is classified as gray desert soil by Chinese scientists and a *Calcaric Fluvisol* according to the FAO. The 0–20 cm depth had the following characteristics: 63 mg kg^−1^ available (mineral) N (potassium chloride extraction), 15 mg kg^−1^ available P (Olsen), and 208 mg kg^−1^ available K (ammonium acetate extraction).

Seeds of the winter wheat cultivar “Xindong 20” were supplied by the Agriculture College of Shihezi University. On the day of sowing, 75 kg ha^−1^ urea (46% N, Sinopec Group) was applied to the soil. The plots were drip irrigated at 10–12 d intervals three times before winter and six times after winter dormancy. Urea was applied via drip irrigation at elongation (45 kg ha^−1^), heading (75 kg ha^−1^), and flowering (120 kg ha^−1^).

The experiment used a randomized block design with three replications. Three P treatments were applied to the plots 160 d after sowing, when about 5% plants of the plants had turned green after dormancy. The P treatments were as follows: 0 kg P ha^−1^ (control, abbreviated P0); 46 kg P ha^−1^ (normal P, abbreviated NP); and 92 kg P ha^−1^ (high P, abbreviated HP). The P fertilizer (triple superphosphate, 45% P) was applied in a 10-cm-deep band between each ridge. The plots were 2.4 × 3 m. Each plot was separated by a 50-cm-wide bare strip.

### Sampling

Grain samples were collected from the middle region of the wheat spikes at 7, 14, 21, 28, and 35 days post anthesis (DPA). The samples were collected from each plot and then pooled to form one sample per treatment. Three subsamples were removed from each composite sample. These subsamples were frozen in liquid N for 5 min and then stored at −80°C for RNA extraction. Another three subsamples were fixed in 4% paraformaldehyde (pH 7.2–7.6) and 0.1% DEPC for histochemical analysis and *in situ* hybridization. The remaining grains were dried at 70°C to constant weight and weighed. Starch granules were isolated from grain (not oven-dried) collected at 35 DPA.

### Isolation of starch granules

Starch granules were isolated using a modified version of the method used by Peng et al. (1999). The embryos were excised from mature wheat grains with a scalpel. The de-embryonated grains were soaked overnight in deionized water at 4°C. Subsequently, the grains were ground in a mortar and pestle with deionized water. The slurry was centrifuged (4,000 g, 10 min). The sediment was treated twice with 80% (w/v) CsCl. The starch was then washed three times with washing buffer (62.5 mmol L^−1^ Tris-HCl, pH = 6.8; 10 mmol L^−1^ EDTA; 4% w/v SDS), three times with deionized water, and finally three times with acetone. The starch samples were then air dried.

### Starch granule morphology

The starch granules were sprinkled onto double-sided conductive adhesive tape attached to aluminum stubs and then coated with gold-palladium (60:40) particles (20 nm particle size) using a sputter coater (Denton Vacuum-Moorestown, NJ, USA). The morphology of the starch granules was examined using a field emission scanning electron microscope (JEOL JFC-1600, Japan) at an accelerating voltage of 5–10 kV.

### Enzyme assays

Total amylase activity was measured as described by Liu (2011). Briefly, fresh, de-embryonated grains (1 g) were ground in 8 mL deionized water. The mixture was placed at room temperature for 15–20 min to extract total amylase. After centrifugation (3,000 rpm, 10 min), 1 mL of soluble starch (1%, w/v) was added to the supernatant (crude enzyme extract) and incubated at 40°C for 5 min. Subsequently, 2 mL of 3, 5-dinitrosalicylic acid reagent (1% w/v 3,5-dinitrosalicylic acid, 30% w/v potassium sodium tartrate, and 0.4 mol L^−1^ NaOH) was added to the mixture before heating in a boiling water bath. Absorbance was measured at 540 nm using a spectrophotometer (Shanghai Precision Scientific Instrument Co., Ltd.722G, China). A standard curve was prepared using malt sugar solutions with concentrations of 0, 0.1, 0.3, 0.5, 0.7, 0.9, and 1.0 mg mL^−1^.

To measure α-amylase activity, β-amylase in the crude enzyme extract was inactivated by heating at 70°C for 15 min. All of the other steps were the same as the assay of total amylase activity described above. Beta-amylase activity was calculated as the difference between total amylase and α-amylase activity.

### Treatment of starch granules with proteolytic enzyme

The isolated starch granules (2 g) were suspended in 40 mL of proteolytic buffer [50 mmol L^−1^ sodium acetate (pH 7.5), 1 mmol L^−1^ calcium chloride, and 0.02% (w/v) sodium azide]. Next, Protease Type XIV [196 units (g starch)^−1^, a mixture of various proteases, Sigma, Lot#051M1894V, USA, source: streptomyces griseus] was added to the suspension before incubating at 4°C for 24 h on a shaking table. After centrifuging at 3,000 g for 20 min, Protease Type XIV-treated starch granules were washed with deionized water and ethanol and then air dried.

### Treatment of starch granules with merbromin and 3-(4-carboxybenzoyl) quinoline-2-carboxaldehyde (CBQCA)

Protease (Type XIV)-treated starch granules were stained with a methanolic solution of merbromin and 3-(4-carboxybenzoyl) quinoline-2-carboxaldehyde (CBQCA) according to Kim and Huber (2008). Merbromin is a non-reactive fluorescent dye that is absorbed onto the surface of the starch granules. Merbromin can be used to highlight external surface of the granules, including channels and cavities connected with the granule's exterior (Huber and BeMiller, 1997). The protein-specific dye CBQCA covalently reacts with primary amines of amino acids, peptides, and proteins under weak basic conditions. The dye, which is fluorescent only after reaction, can be used to highlight the protein network of radially oriented, channel-like structures within starch granules (Han et al., 2005).

### Confocal laser scanning microscopy (CLSM)

After staining with merbromin and CBQCA, the starch samples were transferred to glass slides and then photographed using a Zeiss LSM 510 CLSM system (Zeiss, Oberkoche, Germany). Excitation was achieved with an Argonlaser (488 nm) operating at 30% power. Emission was detected through an LP505 emission filter.

### Detection of wheat starch content

Grain starch content was determined according to the method of Zhao (2005), with three replications. Oven-dried grain was ground using a pulverizer (Shanghai Jiading Grain and Oil Instrument Co., Ltd. JFSD-70, China). Ten milligrams of the powder was transferred into centrifuge tubes and then blended with 100 μL of ethanol and 900 μL of 1 mol L^−1^ NaOH. The mixture was heated in a boiling water bath for 10 min. After centrifugation (800 rpm, 15 min), 500 μl of the supernatant was diluted 200 times with distilled water, 1 mL of 1 mol L^−1^ acetic acid and 1 mL iodine reagents (0.2% I_2_, 2% KI, w/v) were added, color development conditions were room temperature for 10 min, absorbance was measured at 620 nm with a spectrophotometer (Shanghai Precision Scientific Instrument Co., Ltd. 722G, China). A standard curve was prepared using soluble starch solutions at 0, 20, 40, 60, and 80% (w/v).

### Enzymatic hydrolysis of the starch granules

Alpha-amylase and amyloglucosidase digestion was conducted according to Tang et al. (2002). After isolation, the starch granules (25 mg) were suspended in 1 mL sodium acetate solution (0.1 mol L^−1^, pH 4.8) containing 360U α-amylase (Sigma, A4551, USA) or 50U amyloglucosidase (Sigma, A7420, USA). The samples were incubated on a shaking incubator (200 rpm, 37°C, 72 h). Next, 50 μL of 1 mol L^−1^ HCl was added to the samples. The reaction was stopped by adjusting the pH to 7 using 1 mol L^−1^ NaOH.

The extent of starch degradation was determined by measuring the concentrations of reducing sugar produced by starch hydrolysis. Reducing sugar concentrations were determined using a modification of the method described by Bernfield (1951). After centrifuging at 1,500 g for 10 min, 0.1 mL of DNS reagent (0.63% w/v 3,5-dinitrosalicylic acid, 0.524 mol L^−1^ NaOH, 18.5% w/v NaKC_4_H_4_O_6_·4H_2_O, 0.5% w/v phenol, 0.5% w/v NaHSO_3_) was added to 0.1 mL of supernatant. The mixture was heated in a boiling water bath for 5 min. The absorbance was measured at 540 nm with a spectrophotometer (Shanghai Precision Scientific Instrument Co., Ltd.722G, China). A standard curve was prepared at using glucose at concentrations of 0, 100, 200, 300, 400, 500, and 600 μg mL^−1^.

### Detection of relative expression of genes involved in starch biosynthesis and degradation

#### Designation of primers

The primers of *AGP1, AGP2, SS1, SS2, SS3, SS4, GBSS1, GBSS2, SBE1, SBE2A, SBE2B, ISO1, AMY1, AMY2, AMY3, AMY4, BAM1, BAM2, BAM3, BAM4, BAM5, BAM6*, and *BAM7* were designed using Primer Premier 5.0 software according to sequences published in the National Center for Biotechnology Information (NCBI). The primers were synthesized by Sangon Biotech (Shanghai) Co., Ltd. The reference control was wheat *ACTIN* gene. The specificity of the primers was tested and the PCR conditions were optimized using gradient PCR and agarose gel electrophoresis (Bio Rad, Power Pac 300, USA). The primer sequences are presented in Supplementary Table 1.

#### RNA extraction and cDNA synthesis

The RNA was extracted from de-embryonated wheat grain using RNAiso Plus (Takara, Cat#9108, Japan) and Fruit-mate (Takara, Cat#9192, Japan) kits according to the manufacturer's instructions. Total RNA quality was tested using agarose gel electrophoresis. First strand cDNA was synthesized using a reverse transcription kit (Tiangen, Cat#KR104-02, China). The cDNA quality was tested by amplifying the wheat *ACTIN* gene.

#### Quantitative real-time PCR

The rt-qPCR reaction solution was prepared with a SYBR Premix Ex Taq Kit (Takara, Cat#RR420A, Japan). The components are presented in Supplementary Table 2. The amplifications of the individual cDNA sequences were detected using real time qPCR (Roche LightCycler 480 II, USA) with three replications.

A mathematical model was used to determine the relative expression of target gene compared with the reference gene. The relative quantification was calculated with the following formulae:

(1) $$\begin{array}{l} \Delta \text{Ct }\left(\text{target gene}\right)=\text{Ct }\left(\text{target gene}\right)-\text{Ct }\left(ACTIN\text{ of the same sample}\right) \end{array}$$

(2) $$\begin{array}{l} \text{Relative quantification}=2^{-\Delta \text{Ct }\left(\text{target gene}\right)} \end{array}$$

The wheat *ACTIN* gene was used as the reference. The gene is the favored reference for studying wheat genes because it is highly conserved in cell integrity, motility, and structure.

### *In situ* localization of *AMY4, BAM1*, and *BAM5* transcripts

#### Synthesis of the probes

The *AMY4, BAM1*, and *BAM5* gene fragments were amplified using PCR. The fragments were collected using an EasyPure Quick Gel Extraction Kit (Transgen, Code #EG101-01, China). The fragments were linked with pEASY-T3 cloning vectors, and then the vectors were transformed to pEASY-T1 competent cells using a pEASY-T3 cloning kit (Transgen, Cat#CT301-1, China). After screening and culturing, the plasmids were isolated using a TIANprep Mini Plasmid Kit (Tiangen, Cat #DP103-02, China) and sequenced by Sangon Biotech (Shanghai) Co., Ltd. Based on the sequencing results, the plasmids were digested with a restriction enzyme, either Nco I (Takara, Code#1160A, Japan, 10U μl^−1^, source: *Escherichia coli* carrying the plasmid encoding Nco I gene) or Pst I (Takara, Code#1073A, Japan, 15U μl^−1^, source: *Escherichia coli* ED8654 carrying the plasmid encoding Pst I gene). The linearized plasmids were used to synthesize antisense and sense probes via *in vitro* transcription. This was performed using a Dig RNA Labeling Kit (Roche, REF11175025910, USA).

The antisense probe sequence of *AMY4* was as follows: 5′-UUGGUUUCCGAUGGUGUUGUCCAAGAACAGGCAGCUCGCAAUGGCGGGAUCAUUAAGAACGGGAGAGAAAUCCUAUUGCAGGCUUUUAAUUGGGAAUCCCAUAAACACAAUUGGUGGAGUAAUUUAGAGGGCAGAGUUGCCGACAUUGCUA-3′.

The antisense probe sequence of *BAM1* was as follows: 5′-ACUCAGGAAUGCAAGGCCUCAUGGCAUCAACAAGAGCGGCCCUCCUGAGCACAAGCUGUUUGGAUUCACCUACCUCCGGCUGUCGAAUCAGUUGGUGGAGGGACAAAACUAUGUCAAUUUCAAGACCUUUGUUGACAGAAUGCAUGCCAACCUGCCUCAUGACCCAU-3′.

The antisense probe sequence of *BAM5* was as follows: 5′-UGAACCGGAACCUGUUCGACGGCGACAACUGGCGACGGUUCGUCGCGUUCGUGAAGACCAUGGCCGACGGCGGCGCGAGGACGGCGCUGCCCAGGUGCGACACUGGGCACUCGGAUCUGUACGUGGGGUUCGUUGA-3′.

#### Paraffin sectioning

The method for making a paraffin section was modified from Ausubel et al. (1995). Wheat grains were crosscut and fixed in 4% paraformaldehyde (pH 7.2–7.6) and 0.1% DEPC under slight vacuum for 4 h at room temperature. The grains were dehydrated in a graded alcohol series (30–100%), and then cleared three times in solutions of alcohol and chloroform with ratios of 3:1, 1:1, and 1:3, each time for 3 h. Finally, the grains were cleared in absolute chloroform for 3 d. The cleared grains were infiltrated with a graded mixture of chloroform and paraffin wax at different temperatures (3:1, 40°C; 1:1, 40°C; 1:3, 45°C), each time for 4 h. Then, the grains were infiltrated in paraffin wax at 55°C either for 3 d (grain collected of 21 DPA and before) or for 5 d (grain collected at 28 and 35 DPA). The infiltrated grains were embedded in paraffin wax and then sectioned into 10–25 μm thicknesses on a microtome (Kedee, 1508A, China). The exposed surfaces of grain collected at 28 and 35 DPA were soaked in DEPC-water for several hours before being sectioning. The sections were then affixed to adhesive microscope slides (Citoglas, REF188105W, China).

#### *In situ* hybridization

The paraffin sections were dewaxed and rehydrated. Then, *in situ* hybridization was performed with an Enhanced Sensitivity ISH Detection Kit I, POD (Boster, MK1030, China). The paraffin sections were observed and photographed using a stereo microscope (Zeiss Discovery V20, Germany).

### Staining of grain median transverse sections with I_2_-KI

Paraffin sections containing wheat grain were stained with I_2_-KI (0.1%) for 5 min after dewaxing and rehydration. The sections were washed with deionized water, and then observed and photographed using a stereo microscope (Zeiss Discovery V20, Germany).

### Statistical analysis and image processing

The data was analyzed by one-way ANOVA using Microsoft Excel and SPSS 13.0 software. Significance comparisons were made by Duncan's multiple range test at *P* < 0.05. Image processing was performed using Adobe Photoshop CS6.

## Results

### Starch content and starch granule morphology

**Figure 1:**
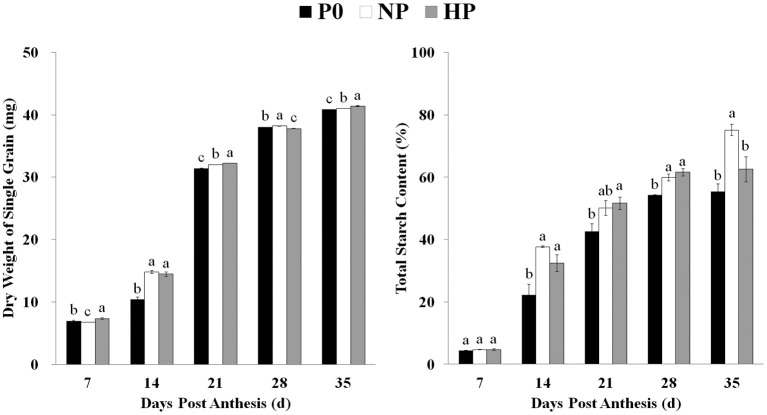
Total starch content and dry weight of single grains in P0, NP, and HP. Values are means ± *S.E*. of three replications. Columns with different letters are significantly different at *p* < 0.05. P0: 0 kg P ha^−1^; NP: 46 kg P ha^−1^; HP: 92 kg P ha^−1^.

Grain weight increased across time and was significantly enhanced by P application (Figure 1). Similarly, the total starch content in the grain was very low during the early grain-filling stage and then increased with grain development. Beginning at 14 DPA, total starch contents were significantly greater in HP and NP than in P0 (note the difference between NP and P0 was not significant on 21 DPA). The grain also matured earlier in HP and NP than in P0 (Supplementary Figure 1).

**Figure 2:**
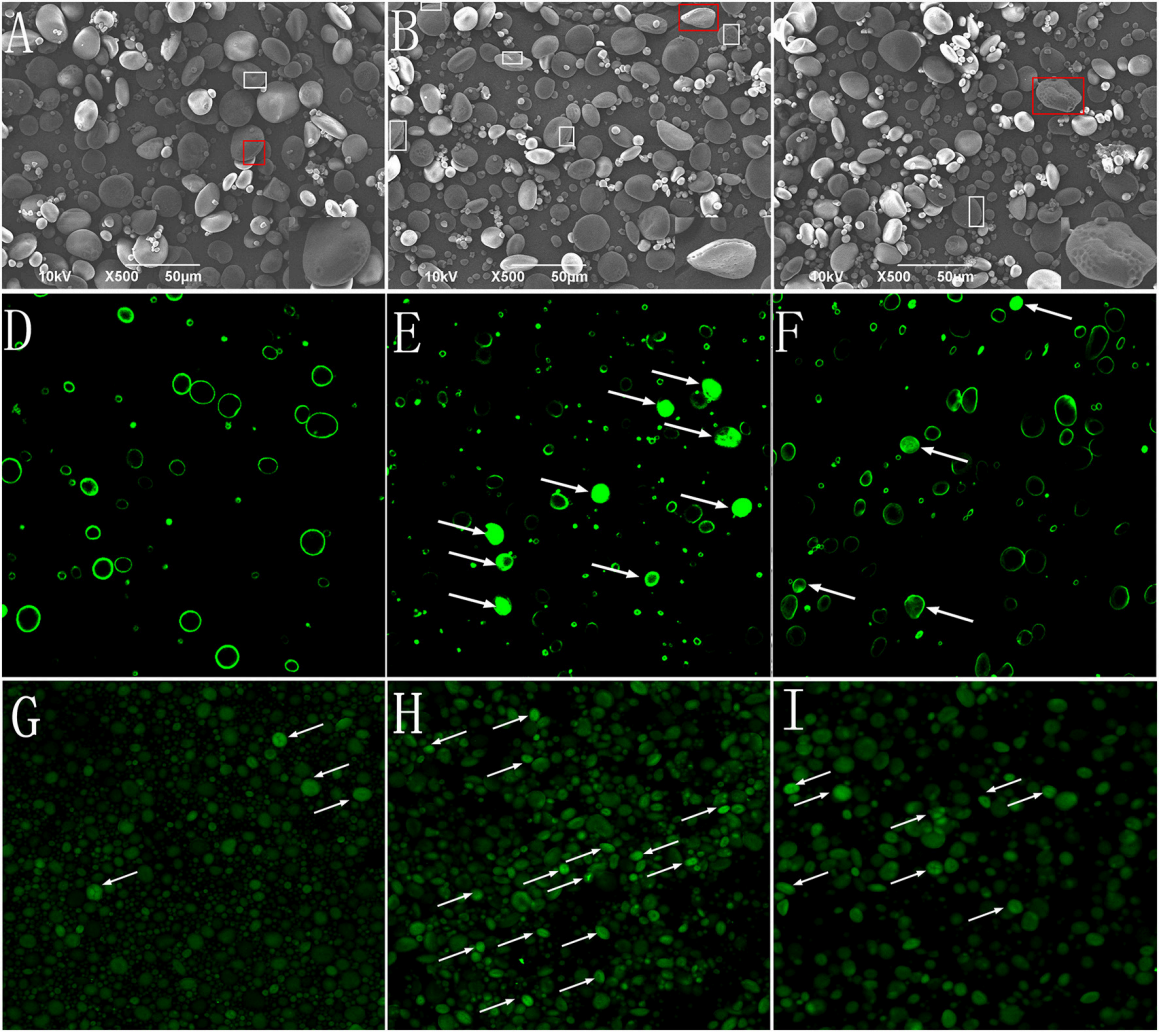
Starch granules isolated from mature wheat seeds (35 days post anthesis) and observed using SEM (×500 magnification) and CLSM (×400 magnification). Starch granules were isolated from the wheat grain in the 0 kg P ha^−1^ (P0) treatment **(A,D,G)**, 46 kg P ha^−1^ (NP) treatment **(B,E,H)**, and 92 kg P ha^−1^ (HP) treatment **(C,F,I)**. **(A–C)**, starch granules observed using SEM; **(D–F)** starch granules stained with merbromin and then observed using CLSM; **(G–I)** starch granules stained with CBQCA and then observed using CLSM. The “pinholes” along the equatorial grooves and on flat surfaces of the granules were visualized with box. The magnified insets (×2,000) **(A–C)** were the starch in red box. Arrows indicate short channels and/or cavities (connected to the exterior by channels) **(E,F)** and radially oriented, channel-like, protein networks **(G–I)** within the granules.

The morphological characteristics of the starch granules was observed using SEM (Figures 2A–C). The A-type granules were disk-shaped with diameters >10 μm. The B-type granules were spherical with diameters <10 μm. The “pinholes” along the equatorial grooves of the granules and on their flat surfaces were more obvious in NP than in HP and P0.

To study the effects of P fertilizer on the micro-structure of starch granules, protease (Type XIV) -treated granules were stained with methanolic merbromin and CBQCA and then visualized by CLSM. The results showed that P fertilizer caused substantial changes in the histochemical patterns of the starch granules. Fluorescence was clearly visible and strong in large areas of many starch granules in NP (Figures 2D–F). In contrast, the P0 samples exhibited only faint fluorescence at the equatorial regions. The fluorescence of starch granules in HP was intermediate between NP and P0. The CBQCA staining (Figures 2G–I) patterns were similar those of membromin. These results suggested that P application influenced, presumably enhanced the pits and channels within starch granules.

### Reducing sugars from starch granules after exogenous enzymatic hydrolysis

**Figure 3:**
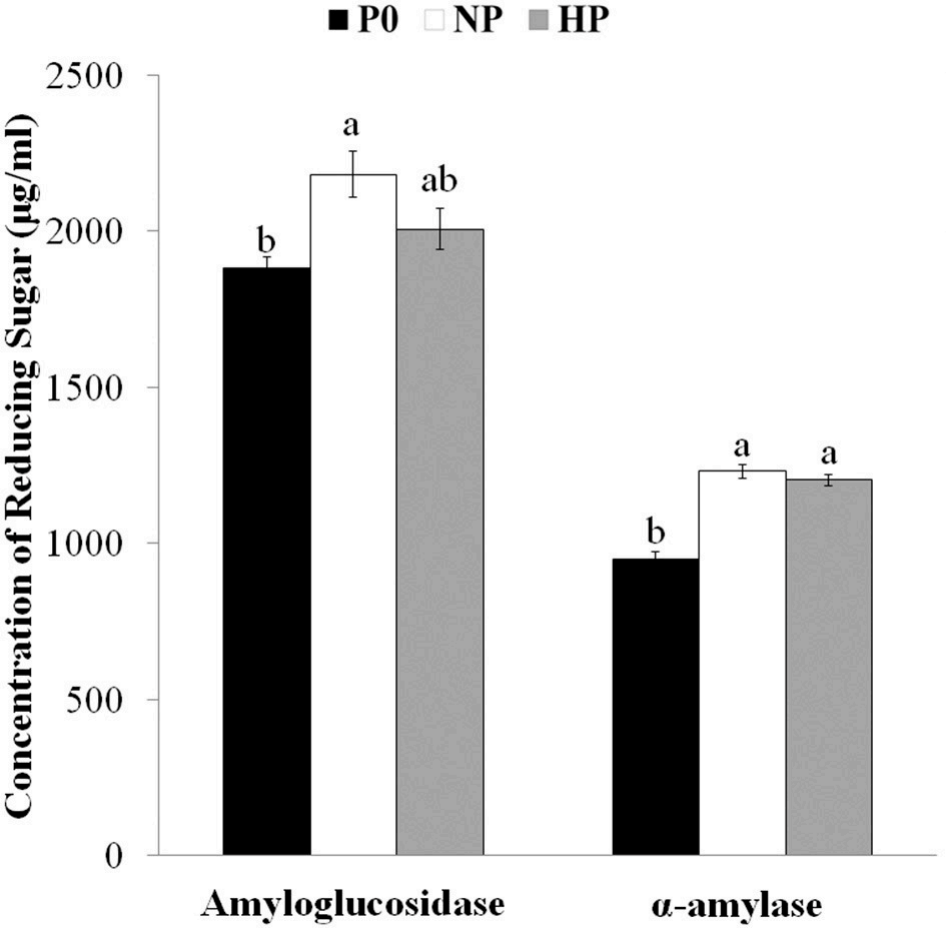
Concentrations of reducing sugars after exogenous enzymatic hydrolysis of starches isolated from mature wheat grain (35 days post anthesis) grown with different P fertilizer amounts. Values are means ± *S.E*. of three replications. Columns with different letters are significantly different at *p* < 0.05. P0: 0 kg P ha^−1^; NP: 46 kg P ha^−1^; HP: 92 kg P ha^−1^.

Drought-induced microstructural changes to starch granules may facilitate the transfer of chemicals (water, enzymes, and acid) into the matrix of the starch granule and accelerate hydrolysis (Li et al., 2015). To ascertain whether the effects of P application were similar to those of drought, we measured the amounts of reducing sugars released from granules after hydrolysis for 72 h with amyloglucosidase and α-amylase. Reducing sugar concentrations after digestion were greater in HP and NP than in P0 (Figure 3, note the difference was not significant between HP and P0 in the amyloglucosidase treated samples). Together with the SEM and CLSM images, this result suggested that P fertilizer enhanced the pits and channels in starch granules and increased the starch surface area available for hydrolysis reactions.

### Patterns of α-amylase and β-amylase activities during grain filling

**Figure 4:**
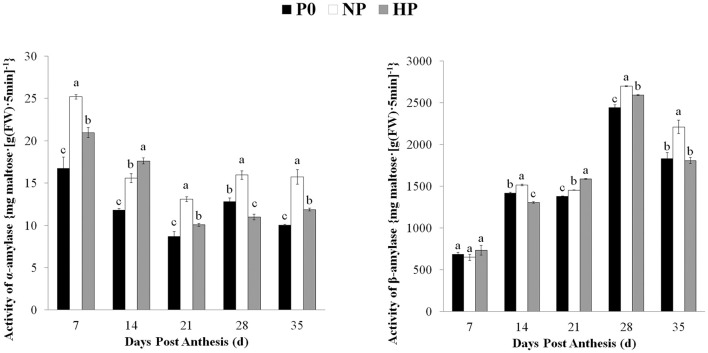
Activities of α- and β-amylase in wheat endosperm as affected by P fertilizer application. Values are means ± *S.E*. of three replications. Columns with different letters are significantly different at *p* < 0.05. P0: 0 kg P ha^−1^; NP: 46 kg P ha^−1^; HP: 92 kg P ha^−1^.

The α- and β-amylase activities in the grain varied depending on sampling time and P treatment (Figure 4). The β-amylase activity was much higher than the α-amylase activity. The α-amylase activity under three treatments was gradually decreased from 7 to 21 DPA and then remained steady. The α-amylase activity was significantly greater in NP than in HP at 7, 21, 28, and 35 DPA. The P0 treatment had the lowest α-amylase activity among the treatments (except for 28 DPA). The β-amylase activity in all three P treatments gradually increased from 7 to 28 DPA and then declined. The β-amylase activity was significantly greater in HP and NP than in P0 at 21 and 28 DPA; however there was no difference between HP and P0 at 35 DPA.

### Patterns of expression of genes involved in starch synthesis and degradation during grain filling

**Figure 5:**
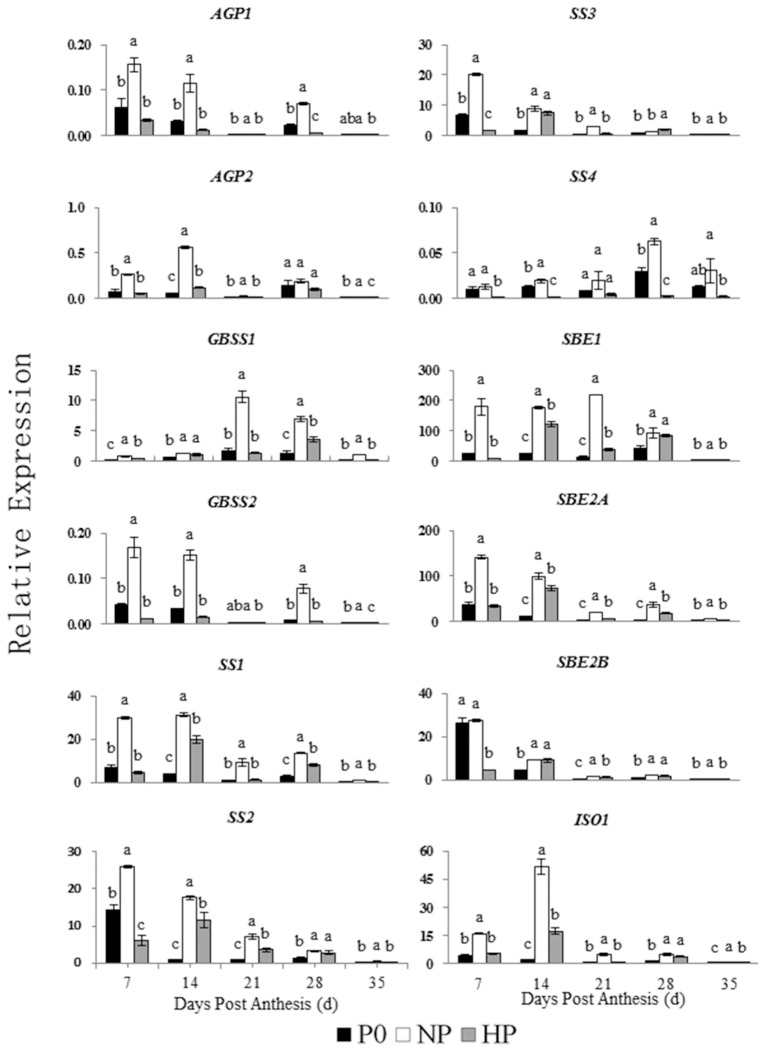
Relative expression of genes involved in starch synthesis in developing wheat endosperm. Values are means ± *S.E*. of three replications. Columns with different letters are significantly different at *p* < 0.05. P0: 0 kg P ha^−1^; NP: 46 kg P ha^−1^; HP: 92 kg P ha^−1^.

**Figure 6:**
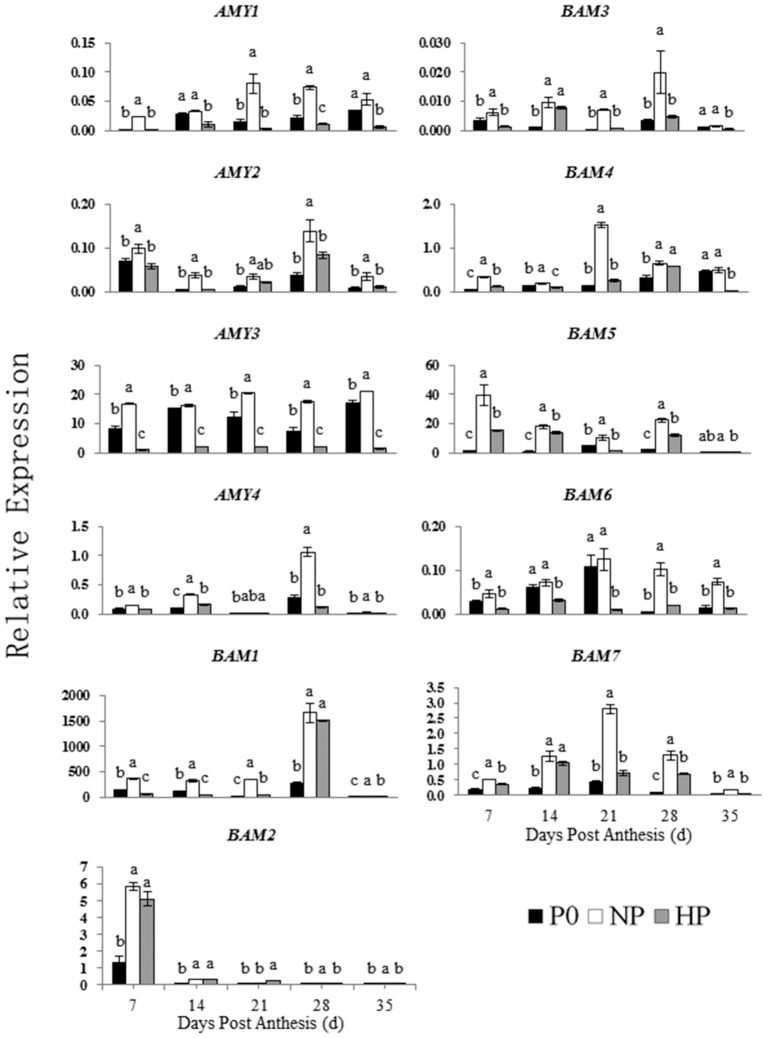
Relative expression of genes involved in starch degradation in developing wheat endosperm. Values are means ± *S.E*. of three replications. Columns with different letters are significantly different at *p* < 0.05. P0: 0 kg P ha^−1^; NP: 46 kg P ha^−1^; HP: 92 kg P ha^−1^.

The relative expressions of genes involved in starch synthesis and degradation are shown in Figures 5, 6. The expression patterns of both *AGP1* and *AGP2* were similar across time in all three P treatments. The NP treatment had the highest *AGP1* and *AGP2* expression at 7 and 14 DPA.

The expression pattern of *GBSS1* was different that of *GBSS2*. The *GBSS1* transcripts were most abundant at 21 and 28 DPA, whereas the *GBSS2* transcripts were most abundant at 7 and 14 DPA. The NP treatment had the highest *GBSS1* expression (at 21 DPA) and the highest *GBSS2* expression (at 7 DPA).

In NP, the *SS1, SS2*, and *SS3* transcripts were greatest at 7 and 14 DPA and then decreased across time. In contrast, the *SS4* transcripts remained steady between 7 and 21 DPA and then increased significantly at 28 DPA. In HP, the *SS1, SS2*, and *SS3* transcripts were gradually increased from 7 to 14 DPA, whereas *SS4* showed little expression on any sample date.

Among the genes encoding starch branching enzyme (*SBE1, SBE2A*, and *SBE2B*), *SBE1* transcripts were most abundant. In NP, *SBE1* transcript abundance was greatest at 21 DPA, whereas the transcript abundances of *SBE2A* and *SBE2B* were both greatest at 7 DPA and then decreased. In HP, the transcripts of *SBE1, SBE2A*, and *SBE2B* were gradually increased from 7 to 14 DPA then decreased during the remaining time.

The NP and HP treatments generally upregulated the *ISO1* transcripts, especially at 14 DPA. The NP treatment upregulated the *AMY1* transcripts compared with P0, with peak expression at 21 DPA. In contrast, HP downregulated *AMY1*. The transcript levels of *AMY2* were significantly greater in NP than in P0 and HP. There was no significant different in *AMY2* expression between P0 and HP between 7 and 35 DPA. The relative expression of *AMY3* in both P0 and NP was high and significantly greater than that in HP between 7 and 35 DPA. The relative expression of *AMY4* increased suddenly at 28 DPA in all three P treatments. The *AMY4* expression was greater in NP than in P0 and HP.

The seven *BAM* genes were differentially expressed among the P treatments. The *BAM* transcript levels were greater in NP than in P0 and HP. In NP and HP, *BAM1* was mostly highly expressed at 28 DPA, whereas *BAM2* was most highly expressed at 7 DPA. Of the expression patterns of *BAM3* and *BAM5* were almost identical both in NP and in HP. However, *BAM3* and *BAM5* genes were weakly expressed in P0. The transcript pattern of *BAM4* significantly varied among the P treatments. The *BAM4* expression at 7 and 14 DPA was less than that on the other sample dates. The transcript patterns of *BAM6* and *BAM7* were similar. The NP treatment upregulated both genes, with relative gene expression reaching a maximum at 21 DPA. The HP treatment downregulated *BAM6* but upregulated *BAM7* compared with P0.

These results indicated that 12 genes involved in starch synthesis and 11 genes involved in starch degradation were expressed in the developing wheat grains. Furthermore, the P treatments significantly influenced the expression patterns of these genes. Compared with P0 and HP, NP upregulated genes encoding starch synthesis enzymes (especially during early to mid-grain filling) and starch degradation enzymes (especially during mid- and late-grain filling).

### Spatial profiling of transcripts of *AMY4, BAM1*, and *BAM5* during grain filling

**Figure 7:**
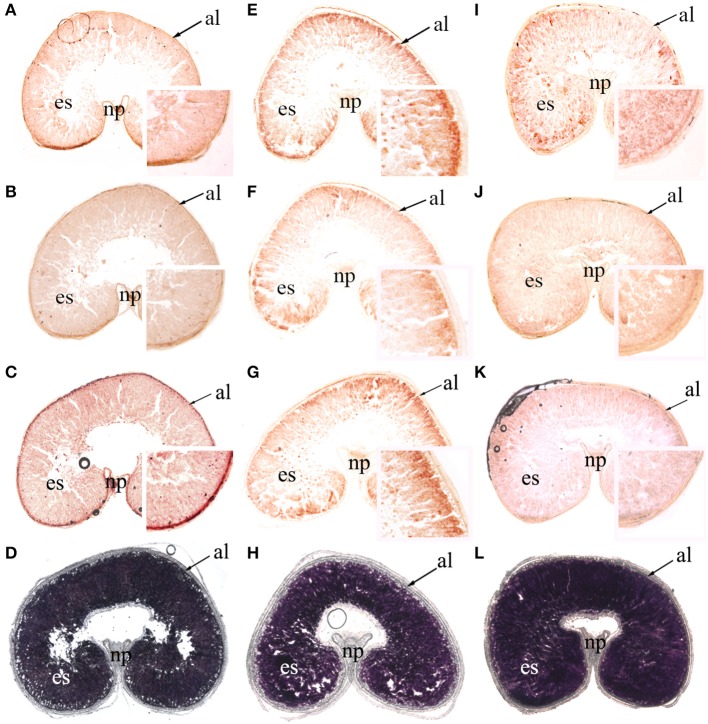
*In situ* localization of *AMY4, BAM1*, and *BAM5*t ranscripts and starch accumulation in wheat caryopses at 28 days post anthesis (×25, magnified insets ×150). Hybridization sites of *AMY4* **(A,E,I)**, *BAM1* **(B,F,J)**, and *BAM5* **(C,G,K)** transcripts were visualized as reddish-brown signals in median transverse sections of wheat grains in the 0 kg P ha^−1^ (P0) treatment **(A–C)**, 46 kg P ha^−1^ (NP) treatment **(E–G)**, and 92 kg P ha^−1^ (HP) treatment **(I–K)**. Starch granules were stained with I_2_-KI in median transverse sections of wheat grains in P0 **(D)**, NP **(H)**, and HP **(L)**. al, aleurone; es, endosperm; np, nucellar projection. The thickness of these sections was 20 μm.

As mentioned previously, β-amylase activity was much greater than α-amylase activity, and the transcription levels of *BAM1* and *BAM5* were the highest among the seven *BAM* genes. In addition, the relative expression of *AMY4* increased sharply to high levels during late grain filling. For these reasons, *AMY4, BAM1*, and *BAM5* mRNA were localized using *in situ* hybridization (anti sense: Figure 7, Supplementary Figures 2, 3; sense control: Supplementary Figures 4–6). Starch accumulation in wheat caryopses (Figures 7D,H,L; Supplementary Figures 3D,H,L) indicated that the cavity in the ventral groove of caryopses was an intrinsic characteristic of wheat grains. *AMY4, BAM1, and BAM5* transcripts were detectable in both the pericarp and early endosperm at 7 DPA in all three P treatments (Supplementary Figure 2). In P0, *AMY4, BAM1*, and *BAM5* transcripts were detected in the entire endosperm from 7 to 35 DPA (Figures 7A–C, Supplementary Figures 3A–C). In NP and HP, *AMY4, BAM1*, and *BAM5* transcripts had accumulated at the endosperm border at 28 DPA (Figures 7E–G,I–K) and at 35 DPA (Supplementary Figures 3E–G,I–K). However, the relative expression of *AMY4, BAM1*, and *BAM5* at the edge of the endosperm was greater in NP (Figures 7E–G, Supplementary Figures 3E–G) than in HP (Figures 7I–K, Supplementary Figures 3I–K). This result showed that P fertilizer increased the transcript abundance of amylase genes at the edge of the endosperm. This phenomenon was more pronounced in NP than in HP.

## Discussion

Wheat endosperm is the main tissue for biosynthesis and accumulation of starch. The A-type and B-type starch granules in mature wheat endosperm display bimodal distribution (Evers, 1971). Based on anatomical studies, Parker (1985) reported that A-type starch granules in wheat were initiated between ~4 and 14 DPA. The B-type starch granules appeared from about 14 DPA until grain maturity. Previous research in our laboratory showed that P fertilizer significantly influenced the ratio of A/B starch granules and the average diameter of the granules (Li et al., 2013). However, the SEM images in this study showed that P fertilizer did not cause significant changes in the shape of the starch granules. Overall, these results indicate that P application may affect the timing and development of A- and B-type starch granules in wheat.

Analysis of the expression patterns of genes involved in starch synthesis is important to understand the mechanism of starch biosynthesis. A previous study showed that AGPase is less sensitive to 3-PGA and inorganic P in cereal endosperm than in other tissues (Gómez-Casati and Iglesias, 2002). This suggests AGPase activity may be controlled at the transcriptional level in endosperm. McCue et al. (2002) found that GBSS I may control starch synthesis at the transcriptional and post-transcriptional level. Wang et al. (2014) studied the relationships among starch accumulation, the activities of key enzymes, and gene expression in wheat endosperm. The results indicated the amylose, amylopectin, and total starch accumulation rate were significantly and positively correlated with the activities of SBE, SSS, and GBSS. The SBE, SSS, and DBE may control starch synthesis at the transcriptional level, whereas GBSS1 may control starch synthesis at the post transcriptional level.

Phosphorus increases photosynthetic rates and promotes post anthesis dry matter accumulation (Zhu et al., 2012). Both of these factors play a vital role in starch biosynthesis and accumulation. In the present study, P application increased the expression of genes involved in starch synthesis, and these increases in gene expression coincided with greater starch content. This indicated that P application promoted starch biosynthesis not only by increasing photosynthetic rates to produce more substrate, but perhaps also by controlling starch synthesis at the transcriptional level. However, excess P application may also enhance respiration, which could increase sugar and energy loss (Shen, 2001). It is well-known that P application can cause earlier seed maturity, and we observed that the grain was darker (or more yellow) in HP than in NP and P0 at 35 DPA (Supplementary Figure 1). This indicates that the grain filling stage in HP was shorter than that in the other two treatments. This perhaps is one reason why HP had less starch accumulation than NP.

Whan et al. (2014) studied α-amylase levels in wheat grain and suggested the endosperm-specific over-expression of *AMY3* resulted in an increase in total α-amylase activity in harvested wheat grain. However, increased α-amylase activity did not significantly influence starch content or composition. As mentioned previously, seed maturity can be promoted by P application (Supplementary Figure 1). In certain varieties of wheat, triticale, and barley, amylase activity always increases with grain maturity (Lindblom et al., 1989; Mares and Oettler, 1991; Radchuk et al., 2009). Thus, it is possible that increased amylase activity in this study resulted from early maturity induced by P application. On the other hand, the increased expression of amylase genes in NP and enhanced amylase activity suggested that P may also control starch degradation at the transcriptional level. Whan et al. (2014) observed that enhanced amylase activity did not reduce starch content. Therefore, there is no contradiction between high amylase activity and high starch content. Increases in amylase activity may cause variation in the channel structures of starch granules.

The channels and pores within starch granules are intrinsic characteristics of wheat. Using CLSM, Kim and Huber (2008) observed that the channel types are different in A- and B-type granules of wheat starch wheat. External factors [e.g., high temperature (Li et al., 2017) and drought stress (Li et al., 2015)] can enhance the number and size of the channels. In this study, “pinholes” and pits on flat surfaces along the equatorial groove of starch granules were more obvious in NP than in HP and P0. The CLSM images and the enzymatic digestion results also confirm such micro-structural changes. We speculate that the increases in channel size and number enlarge the granule surface area available for hydrolysis reactions, resulting ultimately in the release of more glucose units. Earlier investigations in our laboratory revealed that pit micro-structures affected such starch characteristics as pasting properties, swelling power, solubility capacity, and enzymatic hydrolysis (Li et al., 2013, 2015). On the other hand, P-induced changes in the physicochemical properties of starch are complex, and other factors also have significant influence (e.g., the ratio of A- and B-type granules, as well as their volume, internal structure, and surface area; Hayfa et al., 2009).

Fannon et al. (2004) postulated that in the endosperm of sorghum and maize, amyloplasts contain microtubules which radiate outward from the initiation point of starch granule growth (i.e., granule hilum) to the plastid periphery. The authors speculated that the granule develops around the radially-oriented microtubules, which become channels terminating at the outer surface of starch granules. Therefore, the channels within starch granules are the remnants of amyloplast microtubules. Benmoussa et al. (2010) observed that the protein constituents of channels in maize starch included actin-like and tubulin-like structural proteins, a membrane protein (adenylate translocator, Bt1), and the enzymes involved in starch biosynthesis. Further, Benmoussa et al. (2010) hypothesized that microtubules may possess at least two purposes in amyloplasts and starch granules: (1) they may facilitate starch polymer and granule biosynthesis; and (2) they may function to provide variation in the process of granule degradation during seed germination (Fannon et al., 1992a,b).

We are not aware of any previous studies which have examined the biological importance of pits and channels in wheat starch biosynthesis. Based on previous findings in maize, we hypothesize that P fertilizer promotes the development of amyloplasts and influences the structure of amyloplast microtubules (Benmoussa et al., 2010). The microtubules may provide greater surface area for transporting starch-synthesizing enzymes and substrates needed for starch synthesis into the amyloplast. The hypothesis is supported by the observation that NP significantly increased wheat grain starch content as well as the expression of genes involved in starch synthesis. Furthermore, the observation that starch content was greater in NP than in HP suggests that NP optimized the exchange of substances for starch biosynthesis.

During late grain filling, increasing α-amylase and β-amylase activities may act on the channel ends to form pits on the granule surfaces. A previous study in our laboratory showed that endogenous hydrolysis (seed germination) was increased by P-induced increases in the number of pits and channels in wheat starch granules (Zu et al., 2017). Our observations appear to confirm the proposal by Benmoussa et al. (2010) that channels may influence granule degradation during seed germination. Further study is necessary to test this hypothesis. Such studies may provide significant information about the relationship between P application and starch biosynthesis.

Wheat endosperm cells are differentiated from the meristematic region at the periphery of the endosperm. Therefore, the youngest cells are found at the outer edge of the endosperm and the oldest at the center (Bradbury et al., 1956). During wheat grain development, starchy endosperm initiates a cell death program (Young and Gallie, 2000). In this study, P application increased (i) the expression of amylase genes; (ii) amylase activity; and (iii) *AMY4, BAM1*, and *BAM5* transcript abundance at the periphery of the endosperm at 28 DPA. This indicates that during late grain filling in NP, the meristematic region at the periphery of the endosperm still maintained metabolic activities to support the relatively abundant transcripts of genes involved in starch synthesis and degradation. This explains the increased starch content in NP.

## Conclusions

The results of this study show that P fertilizer significantly altered microstructures in the starch granules. This is important because the channels may provide greater surface area for the transport of starch-synthesizing enzymes and substrates needed for starch synthesis. The study also indicated that P fertilizer significantly affected starch accumulation by influencing the expression of genes related to starch biosynthesis and degradation. Further study is necessary to understand the mechanism by which P influences starch morphology and biosynthesis. Such information may provide information helpful for increasing wheat yield and starch quality. The latter could have important implications for the food industry.

## Author contributions

RZ: Substantial contributions to the acquisition, analysis, and interpretation of data for the work; Drafting the work and revising it critically for important intellectual content; Final approval of the version to be published; Agreement to be accountable for all aspects of the work in ensuring that questions related to the accuracy or integrity of any part of the work are appropriately investigated and resolved. KF, CAL: Substantial contributions to the acquisition, analysis, and interpretation of data for the work; Revising the work critically for important intellectual content; Final approval of the version to be published; Agreement to be accountable for all aspects of the work in ensuring that questions related to the accuracy or integrity of any part of the work are appropriately investigated and resolved. CEL, CYL: Substantial contributions to the conception and design of the work; Drafting the work and revising it critically for important intellectual content; Final approval of the version to be published; Agreement to be accountable for all aspects of the work in ensuring that questions related to the accuracy or integrity of any part of the work are appropriately investigated and resolved.

### Conflict of interest statement

The authors declare that the research was conducted in the absence of any commercial or financial relationships that could be construed as a potential conflict of interest.

## Supplementary material

Supplementary Table 1 Characteristics of primers used to measure differential gene expression using Quantitative PCR.

Supplementary Table 2 Components and volume of real time quantitative PCR reaction solution.

Supplementary Figure 1 Fresh grains at 28 DPA **(A–C)** and 35 DPA **(D–F)** under P0 **(A,D)**, NP **(B,E)**, and HP **(C,F)** conditions. P0: 0 kg P ha^−1^; NP: 46 kg P ha^−1^; HP: 92 kg P ha^−1^.

Supplementary Figure 2 *In situ* localization of *AMY4, BAM1*, and *BAM5* transcripts in wheat caryopses at 7 DPA (×30). Hybridization sites of *AMY4* **(A,D,G)**, *BAM1* **(B,E,H)**, and *BAM5* **(C,F,I)** transcripts were visualized as reddish-brown signals in median transverse sections of wheat grains under the P0 **(A–C)**, NP **(D–F)**, and HP **(G–I)** conditions, respectively. es, endosperm; np, nucellar projection; p, pericarp. P0: 0 kg P ha^−1^; NP: 46 kg P ha^−1^; HP: 92 kg P ha^−1^. The thickness of these sections was 20 μm.

Supplementary Figure 3 *In situ* localization of *AMY4, BAM1*, and *BAM5* transcripts and starch accumulation in wheat caryopses at 35 DPA (×25, the magnified insets × 150). Hybridization sites of *AMY4* **(A,E,I)**, *BAM1* **(B,F,J)**, and *BAM5* **(C,G,K)** transcripts were visualized as reddish-brown signals in median transverse sections of wheat grains under the P0 **(A–C)**, NP **(E–G)**, and HP **(I–K)** conditions, respectively. Starch granules were stained with I_2_-KI in median transverse sections of wheat grains under the P0 **(D)**, NP **(H)**, and HP **(L)** conditions, respectively. al, Aleurone; es, endosperm; np, nucellar projection. P0: 0 kg P ha^−1^; NP: 46 kg P ha^−1^; HP: 92 kg P ha^−1^. The thickness of these sections was 20 μm.

Supplementary Figure 4 Sense control of *AMY4, BAM1*, and *BAM5* transcripts in wheat caryopses at 7 DPA (×25). Sense control of *AMY4* **(A,D,G)**, *BAM1* **(B,E,H)**, and *BAM5* **(C,F,I)** transcripts in median transverse sections of wheat grains under the P0 **(A–C)**, NP **(D–F)**, and HP **(G–I)** conditions, respectively. es, endosperm; np, nucellar projection; p, pericarp. P0: 0 kg P ha^−1^; NP: 46 kg P ha^−1^; HP: 92 kg P ha^−1^. The thickness of these sections was 20 μm.

Supplementary Figure 5 Sense control of *AMY4, BAM1*, and *BAM5* transcripts in wheat caryopses at 28 DPA (×25, the magnified insets × 150). Sense control of *AMY4* **(A,D,G)**, *BAM1* **(B,E,H)**, and *BAM5* **(C,F,I)** transcripts in median transverse sections of wheat grains under the P0 **(A–C)**, NP **(D–F)**, and HP **(G–I)** conditions, respectively. al, Aleurone; es, endosperm; np, nucellar projection. P0: 0 kg P ha^−1^; NP: 46 kg P ha^−1^; HP: 92 kg P ha^−1^. The thickness of these sections was 20 μm.

Supplementary Figure 6 Sense control of *AMY4, BAM1*, and *BAM5* transcripts in wheat caryopses at 35 DPA (×25, the magnified insets × 150). Sense control of *AMY4* **(A,D,G)**, *BAM1* **(B,E,H)**, and *BAM5* **(C,F,I)** transcripts in median transverse sections of wheat grains under the P0 **(A–C)**, NP **(D–F)**, and HP **(G–I)** conditions, respectively. al, Aleurone; es, endosperm; np, nucellar projection. P0: 0 kg P ha^−1^; NP: 46 kg P ha^−1^; HP: 92 kg P ha^−1^. The thickness of these sections was 20 μm.

## Abbreviations

AGPase: adenosine diphosphate glucose pyrophosphorylase
*AGP*: gene encoding AGPase
*AMY*: gene encoding α-amylase
*BAM*: gene encoding β-amylase
CLSM: confocal laser scanning microscopy
DPA: days post anthesis
GBSS: granule-bound starch synthase
*GBSS*: gene encoding GBSS
HP: high phosphorus
*ISO*: gene encoding isoamylase
NP: normal phosphorus
P0: phosphorus application control
*SBE*: gene encoding starch branching enzyme
SEM: scanning electron microscopy
*SS*: gene encoding soluble starch synthase.
