# Supplementary material for: Phosphorus Alters Starch Morphology and Gene Expression Related to Starch Biosynthesis and Degradation in Wheat Grain
Source: Front Plant Sci. 2018 Jan 12;8:2252. doi: 10.3389/fpls.2017.02252 (PMC5770358; doi:10.3389/fpls.2017.02252)
Supplement: Supplementary Table 1 — Characteristics of primers used to measure differential gene expression using Quantitative PCR. [file Table1.DOCX]

**Table1. Characteristics of primers used to measure differential gene expression using Quantitative PCR**

| Target gene | accession number | Forward primers | Reverse primers | Annealing temperature (°C) |
| --- | --- | --- | --- | --- |
| *agp1* | AF244997 | AGAAGCGGCAAGGGAGAC | CCTAATACATCAAGGGAGAAAA | 60 |
| *agp2* | Z21969 | TCAAACAAGGAGGGAGTG | GCAGAGGTTGTCGCAGA | 60 |
| *ss1* | AJ292521 | GCAAAAGGAGAGGAGGGTACA | ACGTATGGTCTTTCGTCATGC | 60 |
| *ss2* | AB201445 | GCTACACCAACTTCTCCCTG | GATGATCTCCACGCCCTTCT | 60 |
| *ss3* | AF258608 | GACATGTGGTTTTGCTTGGTT | AGCCAGCGTATATCAGGTGAG | 60 |
| *ss4* | FN179379 | AGCCCGAGGTCTGGAAACA | TCAGGGAACCAACAACCGT | 62 |
| *gbss1* | AF113843 | GACACTATCGTGGAAGGCAAG | TTGACCATCTCATGGTACGC | 60 |
| *gbss2* | AF109395 | CACAGAATGCCAGAGGCATAG | GAACAGATGGGAATCACTCCA | 60 |
| *sbe1* | AF286317 | ATGTTTGGTGGACATGGAAGA | ACGCGATAGTAAGCCACACAA | 60 |
| *sbe2-a* | Y11282 | GGGTTTAGGTGGTGAAGGCTA | AAATCTACGGCGGCATTTATC | 60 |
| *sbe2-b* | AY740401 | CGTATCTCGGAAACATGAGGA | TCCGAGTCTAAGACCACCTTG | 60 |
| *iso1* | AF438328 | CGAGATCTATGTGGCCTTCAA | GAGAGCGCGATCAGGTAAGT | 60 |
| *amy1* | J01236 | TTCAACTGGGAGTCGTGGAAG | GCATGTAACCTTCGTTGGAGAC | 63 |
| *amy2* | M17128 | TGACTTCGCTAGGGGCTACTC | GTCCACCCAGTTCACCAGATT | 63 |
| *amy3* | FN179391 | TGCAGGGATATGCCTACATCC | TGATTTGCAGCTTGCTCTCG | 60 |
| *amy4* | FN179392 | TGGTTTCCGATGGTGTTGTC | TAGCAATGTCGGCAACTCTG | 62 |
| *bam1* | KC175594 | ACTCAGGAATGCAAGGCCTC | ATGGGTCATGAGGCAGGTTG | 62 |
| *bam2* | FN179394 | GCCGAAAGGTATCAACGAGAA | GGGTTATGAGCCAGGTTAGCA | 60 |
| *bam3* | FN179395 | CGCCCTCACCAACTAA | CCCAAGTCACCCGATC | 60 |
| *bam4* | FN179396 | GAGGGAGTCATGGTGGACGT | CGCACTGGTGGAAGGACAT | 60 |
| *bam5* | FN179397 | TGAACCGGAACCTGTTCGAC | TCAACGAACCCCACGTACAG | 57 |
| *bam6* | FN179398 | ACAGGGAAGGAAGGCGAAAC | GAAATGATCCCATCCTCGAAAT | 60 |
| *bam7* | FN179399 | AAAGAGCGTGTCCTTCGAGG | GCTCTCCAGAAGCACCCAAT | 62 |
| *actin* | DN551593 | GGAAAAGTGCAGAGAGACACG | TACAGTGTCTGGATCGGTGGT | 60 |
