# Supplementary material for: Phosphorus Alters Starch Morphology and Gene Expression Related to Starch Biosynthesis and Degradation in Wheat Grain
Source: Front Plant Sci. 2018 Jan 12;8:2252. doi: 10.3389/fpls.2017.02252 (PMC5770358; doi:10.3389/fpls.2017.02252)
Supplement: Supplementary Table 2 — Components and volume of real time quantitative PCR reaction solution. [file Table2.DOCX]

**Table2** **Components and Volume of Real Time** **Quantitative PCR Reaction Solution**

| Components | Volume (μl) |
| --- | --- |
| cDNA template | 0.5 |
| SYBR Green mix | 5 |
| Forward primer | 0.2 |
| Revise primer | 0.2 |
| ddH_2_O | 4.1 |
