# Supplementary figures and images for: Phosphorus Alters Starch Morphology and Gene Expression Related to Starch Biosynthesis and Degradation in Wheat Grain
Source: Front Plant Sci. 2018 Jan 12;8:2252. doi: 10.3389/fpls.2017.02252 (PMC5770358; doi:10.3389/fpls.2017.02252)

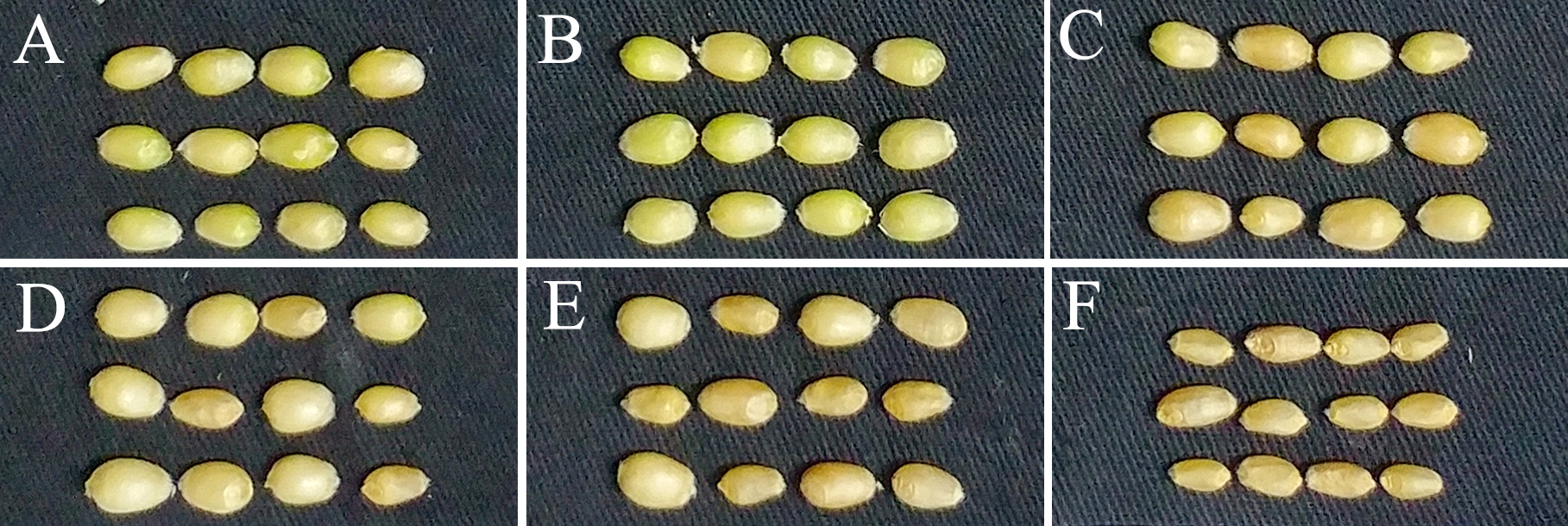

Supplement: Supplementary Figure 1 — Fresh grains at 28 DPA (A–C) and 35 DPA (D–F) under P0 (A,D), NP (B,E), and HP (C,F) conditions. P0: 0 kg P ha−1; NP: 46 kg P ha−1; HP: 92 kg P ha−1. [file Image1.TIF]

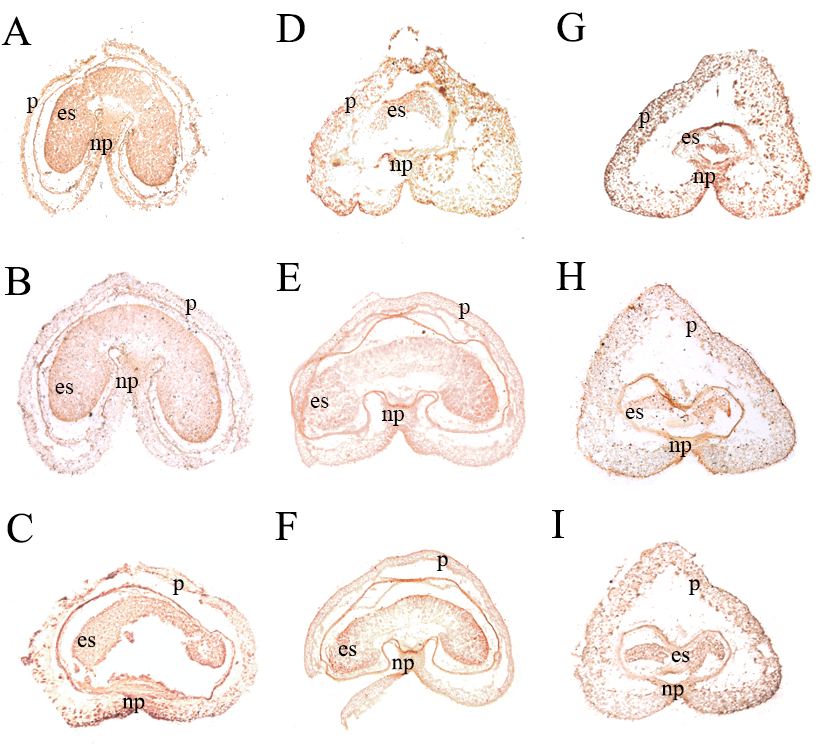

Supplement: Supplementary Figure 2 — In situ localization of AMY4, BAM1, and BAM5 transcripts in wheat caryopses at 7 DPA (×30). Hybridization sites of AMY4 (A,D,G), BAM1 (B,E,H), and BAM5 (C,F,I) transcripts were visualized as reddish-brown signals in median transverse sections of wheat grains under the P0 (A–C), NP (D–F), and HP (G–I) conditions, respectively. es, endosperm; np, nucellar projection; p, pericarp. P0: 0 kg P ha−1; NP: 46 kg P ha−1; HP: 92 kg P ha−1. The thickness of these sections was 20 μm. [file Image2.TIF]

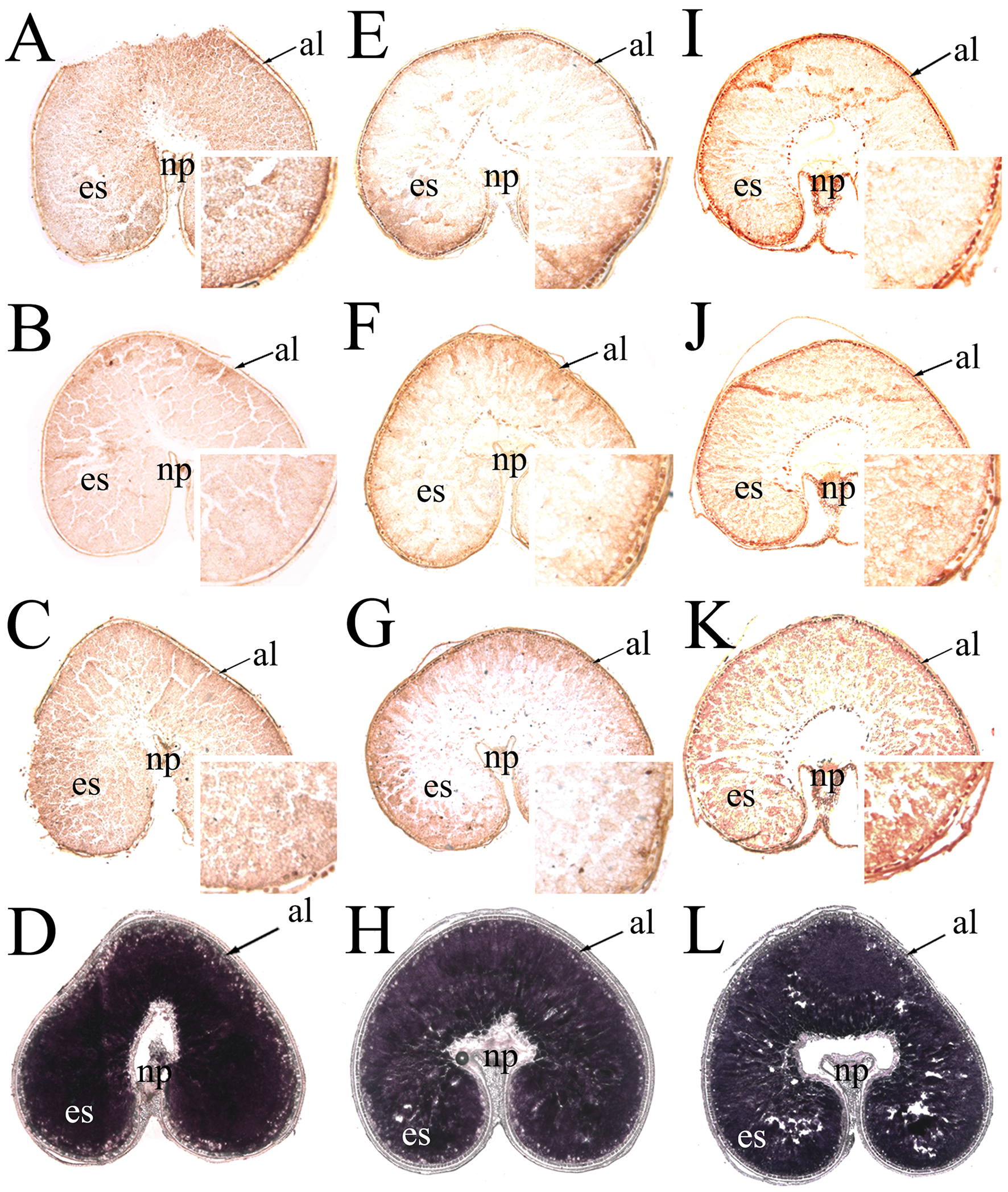

Supplement: Supplementary Figure 3 — In situ localization of AMY4, BAM1, and BAM5 transcripts and starch accumulation in wheat caryopses at 35 DPA (×25, the magnified insets × 150). Hybridization sites of AMY4 (A,E,I), BAM1 (B,F,J), and BAM5 (C,G,K) transcripts were visualized as reddish-brown signals in median transverse sections of wheat grains under the P0 (A–C), NP (E–G), and HP (I–K) conditions, respectively. Starch granules were stained with I2-KI in median transverse sections of wheat grains under the P0 (D), NP (H), and HP (L) conditions, respectively. al, Aleurone; es, endosperm; np, nucellar projection. P0: 0 kg P ha−1; NP: 46 kg P ha−1; HP: 92 kg P ha−1. The thickness of these sections was 20 μm. [file Image3.TIF]

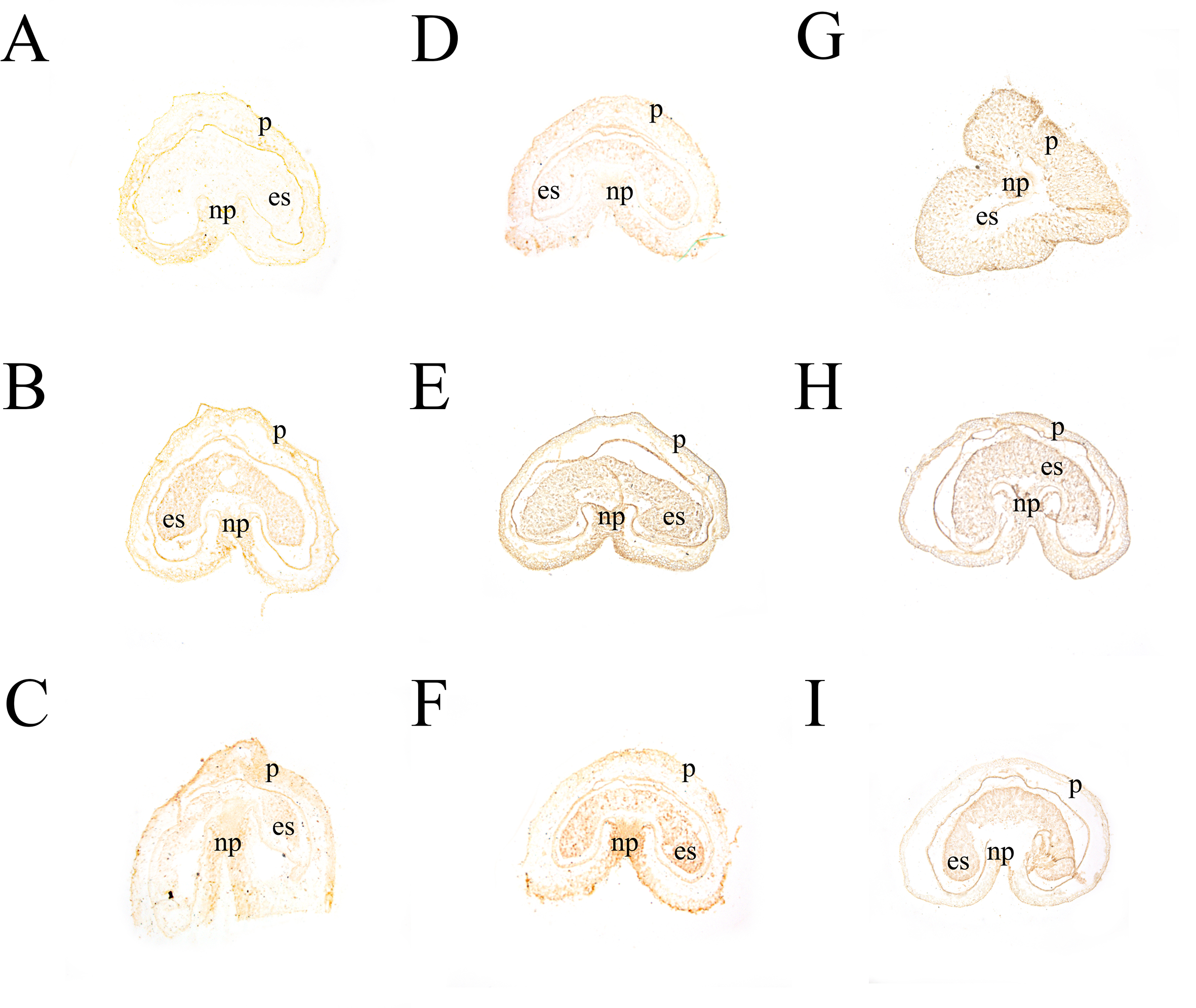

Supplement: Supplementary Figure 4 — Sense control of AMY4, BAM1, and BAM5 transcripts in wheat caryopses at 7 DPA (×25). Sense control of AMY4 (A,D,G), BAM1 (B,E,H), and BAM5 (C,F,I) transcripts in median transverse sections of wheat grains under the P0 (A–C), NP (D–F), and HP (G–I) conditions, respectively. es, endosperm; np, nucellar projection; p, pericarp. P0: 0 kg P ha−1; NP: 46 kg P ha−1; HP: 92 kg P ha−1. The thickness of these sections was 20 μm. [file Image4.TIF]

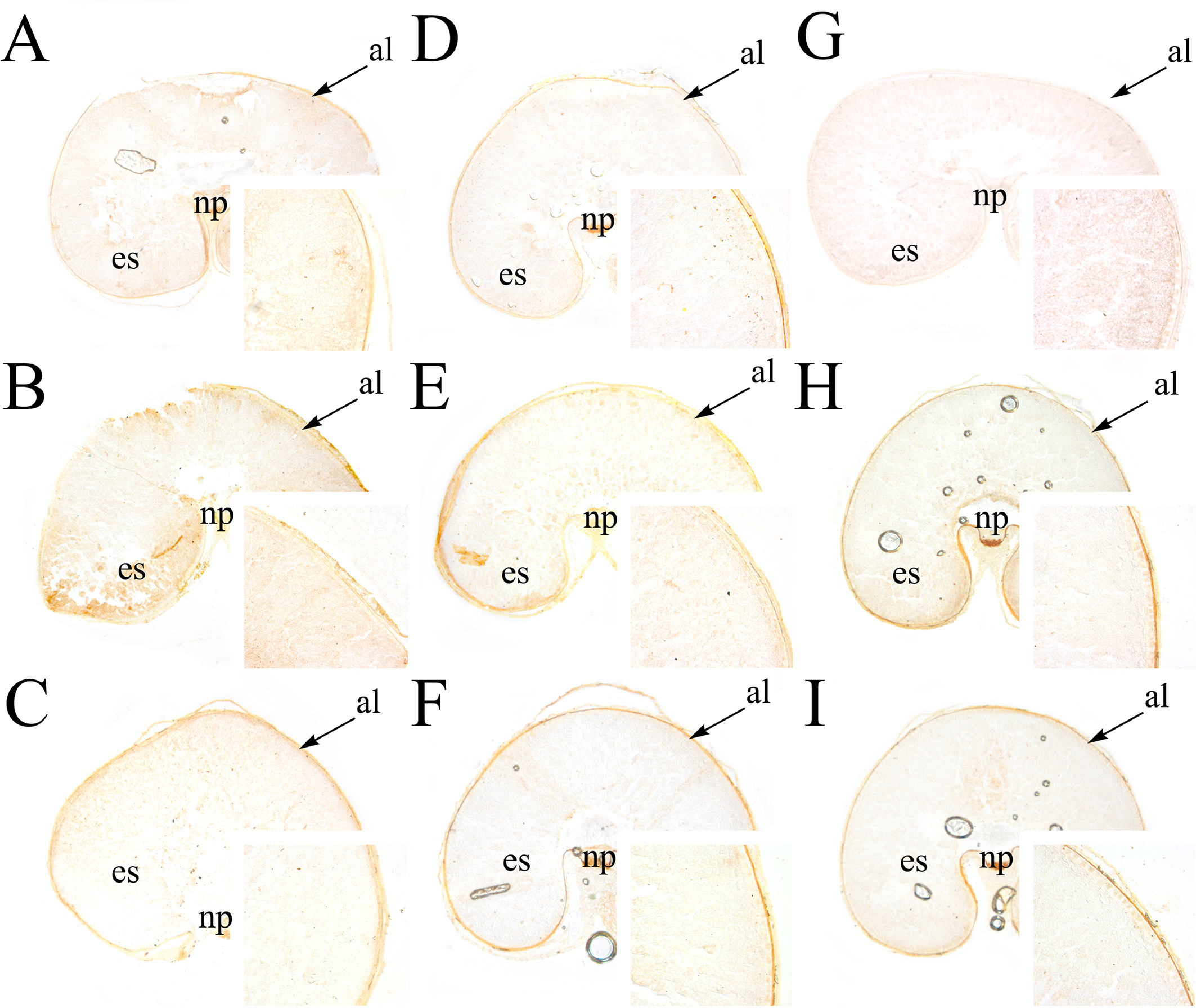

Supplement: Supplementary Figure 5 — Sense control of AMY4, BAM1, and BAM5 transcripts in wheat caryopses at 28 DPA (×25, the magnified insets × 150). Sense control of AMY4 (A,D,G), BAM1 (B,E,H), and BAM5 (C,F,I) transcripts in median transverse sections of wheat grains under the P0 (A–C), NP (D–F), and HP (G–I) conditions, respectively. al, Aleurone; es, endosperm; np, nucellar projection. P0: 0 kg P ha−1; NP: 46 kg P ha−1; HP: 92 kg P ha−1. The thickness of these sections was 20 μm. [file Image5.TIF]

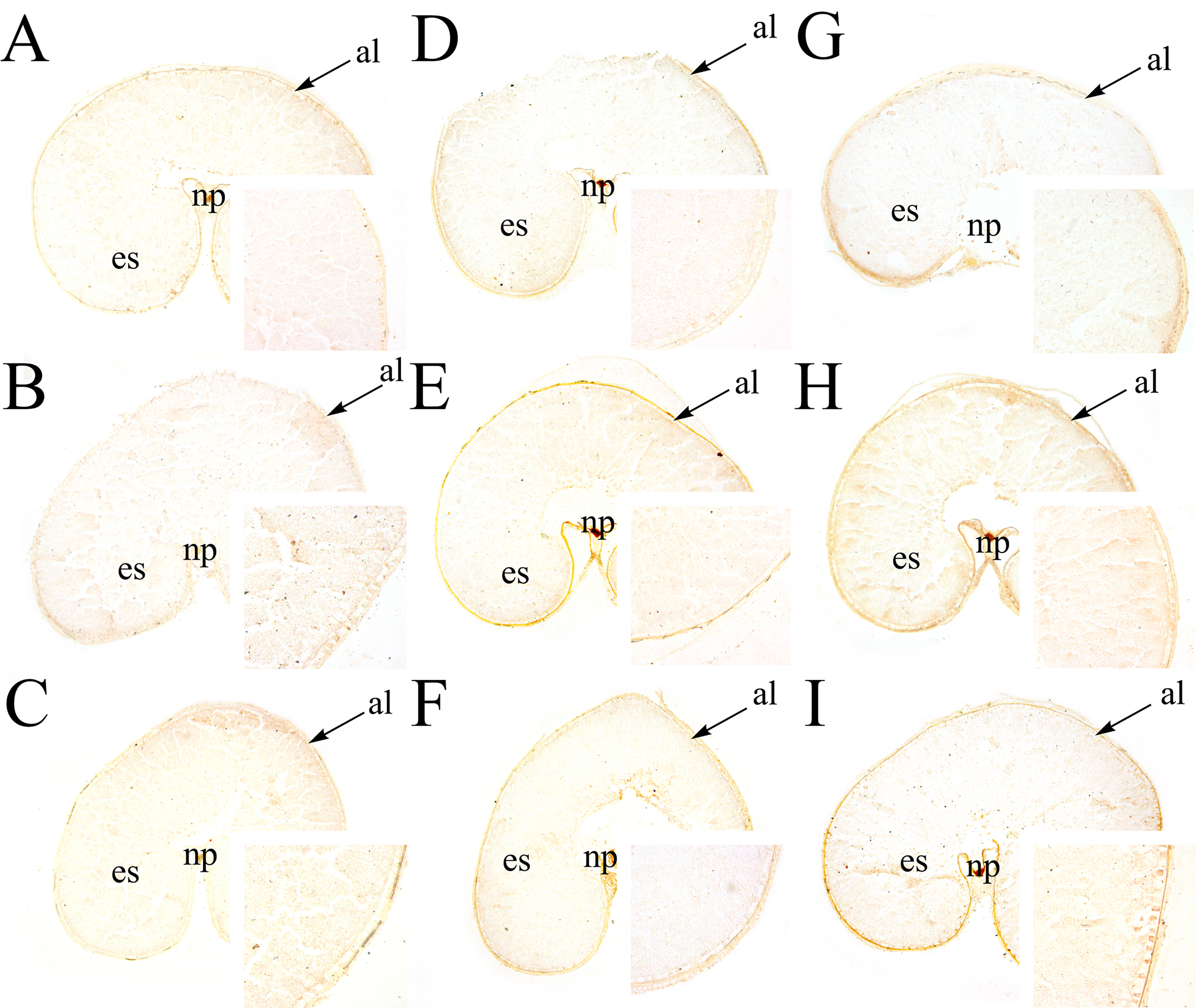

Supplement: Supplementary Figure 6 — Sense control of AMY4, BAM1, and BAM5 transcripts in wheat caryopses at 35 DPA (×25, the magnified insets × 150). Sense control of AMY4 (A,D,G), BAM1 (B,E,H), and BAM5 (C,F,I) transcripts in median transverse sections of wheat grains under the P0 (A–C), NP (D–F), and HP (G–I) conditions, respectively. al, Aleurone; es, endosperm; np, nucellar projection. P0: 0 kg P ha−1; NP: 46 kg P ha−1; HP: 92 kg P ha−1. The thickness of these sections was 20 μm. [file Image6.TIF]
